# Effects of Promotion and Compunction Interventions on Real Intergroup Interactions: Promotion Helps but High Compunction Hurts

**DOI:** 10.3389/fpsyg.2017.00528

**Published:** 2017-04-07

**Authors:** Katy Greenland, Dimitrios Xenias, Gregory R. Maio

**Affiliations:** ^1^School of Social Sciences, Cardiff UniversityCardiff, UK; ^2^School of Psychology, Cardiff UniversityCardiff, UK; ^3^Department of Psychology, University of BathBath, UK

**Keywords:** intergroup contact, compunction, promotion, prejudice/stereotyping, schizophrenia

## Abstract

**HIGHLIGHTS**

We show the promotion intervention has positive effects during intergroup contact, but that high levels of compunction can have negative effects.

Intergroup contact is probably the longest standing and most comprehensively researched intervention to reduce discrimination. It is also part of ordinary social experience, and a key context in which discrimination is played out. In this paper, we explore two additional interventions which are also designed to reduce discrimination, but which have not yet been applied to real intergroup interactions. The promotion intervention encourages participants to relax and enjoy an interaction, while the compunction intervention motivates participants to avoid discrimination. Across two studies, we tested the separate effects of promotion (Study 1) and then compunction (Study 2) on participants' interactions with a confederate whom they believed to have a history of schizophrenia. In Study 1, participants received either a promotion intervention to “relax and have an enjoyable dialogue” or no intervention (control; *n* = 67). In Study 2, participants completed a Single-Category Implicit Attitude Test before being told that they were high in prejudice (high compunction condition) or low in prejudice (low compunction condition; *n* = 62). Results indicated that promotion was associated with broadly positive effects: participants reported more positive experience of the interaction (enjoyment and interest in a future interaction), and more positive evaluations of their contact partner (increased friendliness and reduced stereotyping). There were no effects on participants' reported intergroup anxiety. In contrast, high compunction had broadly negative effects: participants reported more negative experiences of the interaction and more negative evaluations of their contact partner (using the same dependent measures outlined above). In addition, participants in the high compunction condition reported increased intergroup anxiety and increased self-anxiety (anxiety around thinking or doing something that is prejudiced). Participants in the high compunction condition also reported reduced expectancies of self-efficacy (i.e., they were less confident that they would be able to make a good impression).

## Introduction

As our understandings of stereotyping and discrimination have developed, a range of interventions to reduce these social problems have also come about. When discrimination is understood as a consequence of ignorance and unfamiliarity, researchers often focus on intergroup contact (Brown and Hewstone, 2005; Pettigrew and Tropp, 2006). When discrimination is understood as a consequence of unconscious bias, then researchers have focused on motivating participants to change using compunction (Devine et al., 1991; Monteith, 1993). Researchers have also explored the cognitive demands involved in avoiding discrimination, and developed an intervention that attempts to address this phenomenon using promotion (Trawalter and Richeson, 2006). In practice, however, stereotyping and discrimination are driven by a number of processes operating at different levels, and so it makes sense to explore how these interventions work in combination. We suggest that it is particularly important to explore how interventions may impact on intergroup contact: intergroup contact is not only the longest standing and most comprehensively researched intervention in the literature, but it is also a part of ordinary social experience, and a key context in which stereotyping and discrimination are played out.

In this paper, we tested the separate effects of promotion (Study 1) and then compunction (Study 2) on the success of intergroup contact. In both studies, participants interacted with a confederate whom they believed to have a history of schizophrenia. Research shows that attitudes to this group are associated with strong emotional responses that include anxiety and fear (Crisp et al., 2000; Corrigan, 2002). People with a history of schizophrenia are widely seen as dangerous, unpredictable, and unlikely to recover (Crisp et al., 2000; Foster, 2001; see also West et al., 2010).

## Promotion as an intergroup intervention

The promotion intervention (Trawalter and Richeson, 2006) builds on evidence that avoiding discrimination is cognitively demanding. People are often strongly motivated to avoid prejudice, but may feel that they lack the skills to do so (Plant and Devine, 1998, 2003). According to Richeson et al. (Richeson and Shelton, 2003; Richeson and Trawalter, 2005; Trawalter and Richeson, 2006), this means that people often enter intergroup interactions with an avoidance focus (because they are motivated to avoid being prejudiced). Unfortunately, however, Higgins's (1998) Regulatory Focus Theory predicts that an avoidance focus is more cognitively demanding (compared to promotion focus) in that it requires more vigilance and hence more executive attentional capacity. Compared to a promotion focus, avoidance focus can therefore be associated with avoidance behavior, vigilant cognitive processing, and ultimately cognitive depletion and exhaustion (Higgins et al., 1994; see also Plant and Butz, 2006). Theoretically, therefore, an avoidance focus could have (ironically) negative effects on actual intergroup interactions.

Trawalter and Richeson (2006) designed and tested a promotion intervention to replace avoidance with a promotion focus (Higgins et al., 1994). White participants were asked to approach an upcoming interaction with a Black confederate as an “opportunity to have an enjoyable intercultural dialogue” (promotion focus), or to “avoid appearing prejudiced” (avoidance focus), or were given no instructions (control condition). Results indicated that participants in the promotion condition appeared to be more comfortable and experienced less cognitive depletion. Trawalter and Richeson's design did not involve an actual intergroup interaction, but we can extrapolate from their data to make the following predictions.

**Hypothesis 1**. Participants who have experienced a promotion intervention will experience more positive intergroup contact compared to participants who have not had a promotion intervention. Participants often enter contact with a default avoidance motivation (i.e., they wish to avoid appearing to be prejudiced), but this motivation increases the probability of an aversive experience. Replacing default avoidance with a promotion intervention should therefore have a relatively positive effect. This will affect (i) participants' positive evaluation of the contact experience (which we operationalized as measures of enjoyment of the interaction, and a desire for future interaction) and (ii) participants' positive evaluation of their outgroup partner (which we operationalized as perceived friendliness and a reduction in negative stereotyping).

## Compunction as an intergroup intervention

An alternative (and widely researched) intergroup intervention is personal compunction (Devine et al., 1991; Monteith, 1993). Compunction was designed in response to research on the Implicit Association Test (IAT) and unconscious bias (Greenwald et al., 1998; Dovidio et al., 2002; Correll et al., 2007). It involves making participants aware of the gap between their personal standards (in which they are motivated to avoid prejudice) and actual behavior (which may be influenced by unconscious bias). This awareness can be achieved through pencil and paper tasks such as the would/should paradigm (Devine et al., 1991; Monteith and Voils, 1998; Monteith et al., 2010); through false feedback (Monteith, 1993; Monteith et al., 2002); or genuine feedback on a task that is likely to reveal a prejudiced response (Monteith, 1993; Fazio and Hilden, 2001; Gill and Andreychik, 2007; Devine et al., 2012). The IAT is an example of the latter: completing an IAT generates compunction, because participants experience difficultly in controlling their responses and are aware of that difficulty (Monteith et al., 2001; Frantz et al., 2004; Plant and Devine, 2009; Vorauer, 2012).

The compunction intervention therefore makes participants aware of discrepancies between their personal standards and actual behavior, and these discrepancies cause them to experience negative self-directed affect. Theoretically, this negative affect then motivates participants to regulate their subsequent behavior. The dependent measures used in these designs have frequently included changes in implicit or explicit attitudes (Devine et al., 2012), responses to jokes (Monteith, 1993; Monteith and Voils, 1998), or measures of information processing and search (Monteith, 1993; Monteith et al., 2002; Plant and Devine, 2009). Results using these measures have indicated that compunction increased participants' self-monitoring and thereby decreased the impact of implicit associations. However, they have rarely been extended into intergroup interactions. This is important because (as we have outlined above), if self-monitoring is characterized by an avoidance focus, then it may have negative effects during an intergroup contact.

We are aware of only one study that has examined the role of compunction during intergroup contact. Vorauer (2012) explored the effect of completing a race relevant IAT (as opposed to an irrelevant IAT or an explicit measure of racism) on an interracial interaction, and specifically on the metaperceptions of minority participants. Results indicated that when majorities completed a race relevant IAT, they were perceived by their minority partners as less self-disclosing and having less self-efficacy (compared to when participants had not completed an IAT). This study therefore provides some early evidence that the positive effects of compunction may not extend into intergroup contact.

**Hypothesis 2**. Participants who have experienced a high compunction intervention will experience intergroup contact as more negative compared to participants who have had a low compunction intervention. The compunction intervention is designed to generate negative self-directed affect, and this negative affect can impact on participants' experience of contact (Forgas and Locke, 2005). Participants who have had a high compunction intervention will report (i) more negative evaluation of the contact experience (reduced enjoyment of the interaction and reduced desire for future interaction) and (ii) more negative evaluation of their contact partner (judging him as less friendly and negatively stereotyping him more).

## Intergroup contact as an intergroup intervention

Contact is probably the most intuitive and widely researched intervention to improve intergroup relations (Brown and Hewstone, 2005; Pettigrew and Tropp, 2006). It works on the assumption that prejudice is a consequence of ignorance and unfamiliarity that can be dispelled during intergroup dialogue, but perhaps underestimates the ways in which contact can go wrong and the damage that negative contact can have (Vorauer and Sakamoto, 2006; Paolini et al., 2010; Barlow et al., 2012; Graf et al., 2014; but see also Paolini et al., 2014). As we have already outlined, even people who are strongly motivated can inadvertently undermine the success of intergroup contact, either through avoidance motivations or negative self-directed affect (Shelton, 2003; Shelton and Richeson, 2005; see also Vorauer et al., 2009).

We explored two key factors that can undermine the success of intergroup contact: expectancies and intergroup anxiety. Plant et al. (Plant and Devine, 2003; Plant, 2004; Butz and Plant, 2006; Plant et al., 2008) have explored two expectancies that are relevant to intergroup contact: expectancies relating to self-efficacy (i.e., the belief that you can perform in a way that is not prejudiced) and expectancies related to the other (i.e., that your interaction partner is open to meeting you and does not assume that you will be prejudiced). These expectancies can have a positive effect participants' affective and behavioral responses to contact.

In contrast, intergroup anxiety has negative consequences that include stereotyping, negative affect, and a desire to avoid future interactions (e.g., Islam and Hewstone, 1993; Greenland and Brown, 1999; Greenland et al., 2001; Brown and Hewstone, 2005; Turner et al., 2007; but see also Paolini et al., 2016). It can be experienced as “self-anxiety” (a fear that the participant will act or think in a way that makes them appear to be prejudiced) and as “other-anxiety” (a fear that the other represents a risk or a threat; Greenland et al., 2012; see also Plant and Devine, 1998; Stephan and Stephan, 2000). However, it is often measured in the form of “generic anxiety” (measuring the intensity of participants' anxiety without exploring its content, e.g., Stephan and Stephan, 1985; Plant and Devine, 2003).

How might a promotion (i.e., approach) intervention affect expectancies and intergroup anxiety? There is some evidence that expectancies affect approach/avoidance motivations (Butz and Plant, 2006; Plant et al., 2008), but (to our knowledge) no research that has explored the inverse. Similarly, there is some correlational (but no causal) data that links approach/avoidance to self- and other-anxiety. Greenland et al. (Study 4, 2012) reported a positive relation between approach and self-anxiety and a negative relation between approach and other-anxiety. However, there was also a positive relation between self-anxiety and avoidance (which was interpreted by the authors as indicative of freezing; see also Vorauer and Turpie, 2004; Trawalter et al., 2009).

**Hypothesis 3**. We made no specific predictions about the relation between the promotion intervention and expectancies (self-efficacy or of the other) or intergroup anxiety (self-, other-, or generic-intergroup anxiety). Given Hypothesis 1 (that promotion would be associated with a more positive contact experience), we might expect that participants who experience a promotion intervention would also have more positive expectancies and lower intergroup anxiety, but this effect would likely be weak.

In contrast, we might expect a more direct effect of the compunction intervention on both expectancies and intergroup anxiety. The compunction intervention is designed to deliver apparently diagnostic information about participants' prejudice toward the target group. We would expect this to affect participants' expectancies of self-efficacy and self-anxiety.

**Hypothesis 4**. Participants in the high compunction condition will report lower expectancies of self-efficacy and higher levels of self-anxiety (compared to participants in a low compunction condition). The high compunction intervention involves telling participants that they are high in prejudice toward the target group. This should affect participants' expectancies that they can perform in a way that is not prejudiced, and increase their anxiety about acting or thinking in a way that appears to be prejudiced. Given Hypothesis 2 (that high compunction would be associated with a more negative contact experience), we might also expect that participants would have more negative expectancies of the other, and more generic and other-intergroup anxiety.

## The current research

In this paper, we tested the separate effects of promotion (Study 1) and then compunction (Study 2) on participants' interactions with a confederate whom they believed to have a history of schizophrenia. We selected these particular interventions for two reasons. First, because they are well-established in the academic literature; and second, because we had good reason to expect that promotion and compunction might impact on the success of contact. In Study 1, participants received a promotion intervention, in which they were instructed to try to relax and enjoy the interaction. In Study 2, participants received a high compunction intervention, in which they received false feedback indicating that they were high in prejudice toward people with mental health difficulties.

## Study 1

Drawing on Trawalter and Richeson (2006), we evaluated the effects of a promotion focus intervention on an actual interaction with a confederate who participants believed had a history of schizophrenia. Participants engaged in a real interaction in the context of a shared task. Consistent with Trawalter and Richeson (2006), we expected that participants in the promotion condition would have a more positive experience compared to participants in the control condition (Hypothesis 1) but made no specific predictions for expectancies and intergroup anxiety (Hypothesis 3).

### Methods

#### Participants

Sixty-seven British female undergraduate students took part for course credit or payment (we did not conduct an a priori power calculation but rather a rule of thumb of 30 participants per cell, with some latitude for missing data). Participants were between 18 and 25 years of age (*M*_age_ = 19.31, *SD* = 1.33). Eleven participants said that they had a history of schizophrenia or knew someone who did; separate analyses on these participants confirmed that they did not differ from the remainder of the sample and they were therefore included in all analyses.

#### Design

Participants met and worked on a task with a confederate whom they believed to have a history of schizophrenia. Half of the participants were given a promotion-focused instruction (promotion condition), while the other half were given no specific instructions (control). Participants were randomly allocated to condition. The dependent variables were assessed after the manipulation, with some before and some after the interaction. Intergroup anxiety (self-, other-, and generic), and expectancies (of self-efficacy and of the other) were assessed before the interaction. Participants' enjoyment of the interaction, perceptions of the target's friendliness, and stereotyping of the confederate were assessed immediately after the interaction.

#### Procedure and manipulation

When participants arrived in the laboratory, they were informed that they would take part in the “NASA space task,” which would involve working with another person. The next set of instructions was administered via a computer presentation. First, participants were reminded that they would meet someone and work with him on the task. The subsequent slide then stated that the person they would meet had a history of schizophrenia. All participants (i.e., in both promotion and control conditions) saw this slide. The experimental manipulation (which was modeled on Trawalter and Richeson, 2006) followed: participants in the promotion condition read, “This interaction is an opportunity for you to relax and have an enjoyable dialogue. Have fun!” Participants in the control condition read “Please click to continue.”

Participants in both conditions were then instructed to summon the experimenter, who briefly introduced them to the confederate in order to maximize the plausibility of the design. The confederate was male, had no history of mental health problems, and was trained to be pleasant but not overfriendly with participants. Both the experimenter and the confederate were blind to condition, and the same confederate was used for all of the participants.

The participant was taken to an adjacent room, where pre-interaction measures (outlined below) were completed. Participants were then told that they would have one of two roles in the discussion: the generator (who would generate ideas for solving the task), or the responder (who would give feedback and comments to the generator's ideas). A rigged ballot ensured that participants were always the generator. This small modification of the task reduced the contribution of the confederate and thereby increased experimental control.

The participant and the confederate then worked through the NASA space task, which involved ranking 15 items that would be most useful to an astronaut making a 200-mile trip across the surface of the moon (e.g., parachute silk, a magnetic compass, two 0.45 caliber pistols). The confederate followed a flexible script in which he agreed with the first three suggestions made by the participant but then disagreed with the fourth. The confederate was trained with arguments that he might make for or against any of the 15 items.

After a certain point, the experimenter was summoned, and the participant completed the post-interaction dependent measures. This was the end of the study. Participants were debriefed and probed for suspicion using a standard, semi-structured funnel procedure. None of the participants withdrew their data after full disclosure.

#### Pre-interaction dependent measures

Participants completed intergroup anxiety measures after the manipulation and before the upcoming interaction. These included the 20-item Self Other Intergroup Anxiety Scale (SOIAS; Greenland et al., 2012) which distinguishes self-anxiety (anxiety over thinking or doing something that is prejudiced; e.g., “I am anxious about doing something that makes me look prejudiced;” α = 0.87) and other-anxiety (anxiety that the other person might do something to you; e.g., “I am anxious about him being difficult;” α = 0.91), and Plant and Devine's (2003) generic, 4-item, intergroup anxiety measure (e.g., “I will feel uncomfortable when interacting with him;” α = 0.85). The response scale for these measures was anchored at −3 (“Strongly disagree”) and +3 (“Strongly agree”).

Participants also completed two expectancy measures, both adapted from Butz and Plant (2006; Plant et al., 2008). Expectancy of self-efficacy was measured with five items (e.g., “I am confident that I will make a good impression during this interaction;” α = 0.84) and expectancy of the other was measured with six items (e.g., “regardless of my behavior, my interaction partner will view me as prejudiced” (reversed); α = 0.84). Participants responded to both using a 7-point scale anchored at −3 (strongly disagree) and +3 (strongly agree).

#### Post-interaction dependent measures

Two questions served as manipulation checks (e.g., “This interaction was an opportunity for me to relax”). Responses to these items were strongly correlated (*r* = 0.49, *p* < 0.001), and were therefore averaged to form a single indicator. Participants responded to these items using a 7-point scale anchored at 1 (not at all) and 7 (very much).

We asked participants how much they had enjoyed the interaction (three items; e.g., “I found the interaction enjoyable;” α = 0.97) and how much they were interested in a future interaction with the confederate (six items; e.g., “I would be happy to meet with this person again;” α = 0.91; adapted from (Plant and Butz, 2006), Study 2). We also asked participants to rate the confederate's friendliness using five items (e.g., “pleasant” and “unfriendly;” α = 0.82) from Dovidio et al. (2002). Participants were asked to report the extent to which they agreed or disagreed with each of the items using a 7-point scale from 1 (“not at all”) to 7 (“very much”).

Finally, we developed a novel measure of stereotyping. Crisp et al. (2000) suggested that stereotypes of people with schizophrenia are founded on beliefs that they are unpredictable, dangerous, and socially awkward (see also Foster, 2001). Given the nature of the task, we added incompetence to these three factors, yielding a 20-item stereotype measure. Participants were asked to rate the confederate using the 7-point scale from 1 to 7 outlined above. Initial pilot work suggested the presence of one factor, and items loading >0.55 were selected for a final 12 item short form (hard to talk to, uncommunicative, unresponsive, helpless, competent (reverse-coded), capable (r), ineffective, unpredictable, reasonable (r), consistent (r), easy-going (r), and gentle (r). In Study 1, these 12 items loaded onto two factors, such that items on the first factor were positively valenced and items on the second factor were negatively valenced (see Table 1). The two factors were correlated (*r* = 0.56) and there was good internal reliability (α = 0.92). The high factorial coherence and internal consistency of these items led us to derive an overall stereotyping score by averaging across the items. Higher scores indicated negatively valenced stereotyping of the confederate as someone who had a history of schizophrenia.

**Table 1:** **Factor loadings for exploratory factor analysis of schizophrenia stereotyping measure (Study 1) with oblimin rotation**.

	**Positive items**	**Negative items**
Hard to talk to	0.17	**0.76**
Uncommunicative	0.14	**0.78**
Unresponsive	0.23	**0.74**
Helpless	0.09	**0.69**
Competent	−**0.76**	−0.12
Capable	−**0.79**	−0.12
Ineffective	0.24	**0.70**
Unpredictable	−0.21	**0.80**
Reasonable	−**0.94**	0.16
Consistent	−**0.68**	−0.13
Easy-going	−**0.76**	−0.10
Gentle	−**0.70**	−0.01

*Factor loadings > 0.40 are in boldface*.

### Results and discussion

#### Manipulation check

The manipulation check indicated that the promotion focus intervention was successful. Participants said that they relaxed more in the promotion condition compared to the control condition (*M*_promotion_ = 4.47, *SD* = 1.02; *M*_non−promotion_ = 4.05, *SD* = 1.21; *p* < 0.05, *d* = 0.668, η*p*^2^ = 0.090; see Table 2).

**Hypothesis 1**. We predicted that participants in the promotion condition would have a more positive experience compared to participants in the control condition. This was supported by our data: the promotion intervention was associated with a number of positive effects on the post-interaction dependent measures. As shown in Table 2, participants in the promotion condition reported that they enjoyed the interaction more (*M*_promotion_ = 5.82, *SD* = 1.10; *M*_non−promotion_ = 5.04, *SD* = 1.25; *p* < 0.01, *d* = 0.75, η*p*^2^ = 0.101), expressed a greater interest in a future interaction (*M*_promotion_ = 6.06, *SD* = 0.86; *M*_non−promotion_ = 5.32, *SD* = 1.23; *p* < 0.01, *d* = 0.80, η*p*^2^ = 0.112), and said that the target was more friendly (*M*_promotion_ = 6.50, *SD* = 0.47; *M*_non−promotion_ = 6.20, *SD* = 0.64; *p* < 0.05, *d* = 0.56, η*p*^2^ = 0.067). They also stereotyped him less (*M*_promotion_ = 2.45, *SD* = 0.96; *M*_non−promotion_ = 1.98, *SD* = 0.79; *p* < 0.05, *d* = 0.56, η*p*^2^ = 0.067). Cohen's *d* indicated medium to large effect sizes.

**Hypothesis 3**. We made no specific predictions about the relation between promotion and either expectancies or intergroup anxiety. Although it was possible that promotion might be associated with more positive expectancies and lower intergroup anxiety, we expected that this effect would likely be weak. In fact, there were two small effects (Cohen's *d* > 0.20) that did not reach statistical significance (see Table 3). Participants who had had the promotion intervention tended to report lower levels of generic intergroup anxiety in the minutes immediately before the interaction (*M*_promotion_ = −0.75, *SD* = 1.41; *M*_non−promotion_ = −0.29, *SD* = 1.11; *p* = 0.146, *d* = 0.305, η*p*^2^ = 0.032) and more positive expectancies of the other (*M*_promotion_ = 5.90, *SD* = 0.80; *M*_non−promotion_ = 5.69, *SD* = 0.66; *p* = 0.248, *d* = 0.210, η*p*^2^ = 0.020). There were no effects on self- or other-anxiety, or on expectancies of self-efficacy.

**Table 2:** **Contrasting the contact and stereotyping dependent measures by condition (Study 1)**.

**Dependent measure**	**Non-promotion condition mean *(SD)***	**Promotion condition mean *(SD)***	
Manipulation check	4.05 (1.21)	4.74 (1.02)	*t*_(60)_ = 2.43, *p* < 0.05, *d* = 0.668, *ηp^2^* = 0.090
Enjoyed the interaction	5.04 (1.25)	5.82 (1.10)	*t*_(65)_ = 2.68, *p* < 0.01, *d* = 0.75, *ηp^2^* = 0.101
Interest in future interaction	5.32 (1.23)	6.06 (0.86)	*t*_(64)_ = 2.85, *p* < 0.01, *d* = 0.80, *ηp^2^* = 0.112
Friendliness	6.20 (0.64)	6.50 (0.47)	*t*_(65)_ = 2.14, *p* < 0.05, *d* = 0.56, *ηp^2^* = 0.067
Stereotyping	2.45 (0.96)	1.98 (0.79)	*t*_(65)_ = 2.15, *p* < 0.05, *d* = 0.56, *ηp^2^* = 0.067

**Table 3:** **Contrasting the expectancies and intergroup anxiety dependent measures by condition (Study 1)**.

**Dependent measure**	**Non-promotion condition mean *(SD)***	**Promotion condition mean *(SD)***	
Generic intergroup anxiety	−0.29 (1.11)	−0.75 (1.41)	*t*_(65)_ = 1.47, *p* = 0.146, *d* = 0.305, *ηp^2^* = 0.032
Self-anxiety	−0.77 (1.03)	−0.60 (1.00)	*t*_(65)_ = 0.66, *p* = 0.512, *d* = 0.100, *ηp^2^* = 0.007
Other-anxiety	−1.48 (1.16)	−1.64 (1.56)	*t*_(65)_ = 0.52, *p* = 0.604, *d* = 0.081, *ηp^2^* = 0.004
Expectancy of self-efficacy	4.75 (0.92)	4.76 (1.13)	*t*_(65)_ = 0.04, *p* = 0.966, *d* = 0.050, *ηp^2^* = 0.000
Expectancy of the other	5.69 (0.66)	5.90 (0.80)	*t*_(65)_ = 1.17, *p* = 0.248, *d* = 0.210, *ηp^2^* = 0.020

The study aimed to evaluate a promotion-focused intervention on an actual intergroup interaction. Our results revealed overall positive effects of the intervention on the interaction itself, and some weaker effects on the antecedents of positive contact (i.e., expectancies and intergroup anxiety).

## Study 2

The results of Study 1 revealed positive effects of a promotion manipulation during intergroup contact. Study 2 used the same context (interacting with a confederate who apparently had a history of schizophrenia) and the same dependent measures. However, we replaced the promotion intervention with compunction. Participants in both conditions completed the Single Category Implicit Attitude Test (SC-IAT; Karpinski and Steinman, 2006), an adaption of the IAT. They were then given feedback to indicate that they were either high in prejudice toward people with mental health difficulties (high compunction condition) or low in prejudice toward people with mental health difficulties (low compunction condition).

Our manipulation of compunction was therefore slightly different to previous designs which compared participants who had completed a relevant IAT condition with a non-IAT (or non-relevant IAT) control condition (e.g., Monteith et al., 2001; Plant and Devine, 2009; Vorauer, 2012). We used the SC-IAT (rather than an IAT) because of the absence of a relevant outgroup to the category “people with a mental illness.” The conventional IAT requires the pairing of ingroup/outgroup items that was not available for our target group.

Participants in both the compunction and control conditions completed the SC-IAT: the manipulation was contained in the feedback that they had to the task. This was because the SC-IAT used the single category “mental illness” (target words included crazy, mental, and psycho) and could therefore potentially prime a negative stereotype associated with mental illness. This would have generated a potential confound in comparing participants who had/had not completed the SC-IAT. Instead, all of our participants completed the SC-IAT, but we then gave them false feedback that would either confirm their compunction (i.e., that the results showed that they were high in prejudice) or disconfirm (and therefore reduce) their compunction (i.e., that they were low in prejudice). Giving participants neutral or no feedback would not have been effective, since simply completing an IAT has been shown to generate compunction (Monteith et al., 2001; Plant and Devine, 2009).

We expected that participants in the high compunction condition would have a more negative contact experience compared to participants in the low compunction condition (Hypothesis 2), more negative expectancies of self-efficacy, and more self-anxiety (Hypothesis 4). They might also have more negative expectancies of the other and more generic intergroup anxiety.

### Method

#### Participants

Sixty-two British female undergraduate students took part for course credit (as in Study 1, we used a rule of thumb of 30 participants per cell). Participants were aged between 18 and 28 years (*M*_age_ = 18.6, *SD* = 1.33). Twelve participants reported that they knew someone who had had a history of schizophrenia, but further analysis did not reveal any impact of these participants on the results. These participants were therefore included in the analyses.

#### Design

The study was based on Study 1, but with a compunction manipulation rather than promotion. Participants met and worked on a task with a confederate whom they believed to have a history of schizophrenia. Prior to this interaction, all participants had completed a SC-IAT (Karpinski and Steinman, 2006), and been given false feedback on that task. Half of the participants were given feedback to indicate that they were high in prejudice toward people with a mental illness (high compunction condition) and half were given feedback that they were low in prejudice (low compunction condition). Participants were randomly allocated to condition. The dependent variables were the same as Study 1, but with the addition of negative self-affect as a manipulation check.

#### Procedure and manipulation

Participants were told a slightly different cover story compared to Study 1. They were told that the experiment would explore how a social experience might change their performance on a computer-mediated measure of attitudes, and that it was in three parts. First, they would complete an SC-IAT. Second, they would meet and work with another person. Third, they would repeat the SC-IAT to see if their scores had changed (this third part of the experiment did not actually take place).

All of the participants completed an SC-IAT, outlined below. Participants were asked to sort words into three categories as quickly as possible, but using only two keys (“1” and “3” on the number pad). The categories were good (e.g., beautiful, cheerful, friendly), bad (e.g., angry, brutal, dirty), and mental illness (e.g., crazy, mad, insane). The full set of categories and associated words are reproduced in the Appendix 1 (note that we designed the SC-IAT in order to generate plausible feedback for our experimental manipulation rather than to generate data *per-se*. We therefore do not describe any data from the SC-IAT: our focus was on the way the feedback affected participants).

In stereotype-congruent trials, participants used the same key to categorize associated constructs (i.e., “bad” and “mental illness”). In stereotype-incongruent trials, participants used the same key to categorize constructs that were not associated (i.e., “good” and “mental illness”). There were four blocks. Blocks one and three were practice blocks and consisted of 24 trials; blocks two and four were test blocks and consisted of 72 trials. Participants were instructed to respond as quickly as possible, being careful not to make errors. The full task (including practice trials) took ~10 min to complete.

Participants were then told that their results were being computed, and there was a pause of 20 seconds. The next screen contained the manipulation. In the high compunction condition, the feedback slide stated, “your results suggest that you are high in prejudice toward people with mental health difficulties.” In the low compunction condition, the slide stated, “your results suggest that you are low in prejudice toward people with mental health difficulties.”

The rest of the procedure was identical to the schizophrenia condition of Study 1. Participants were reminded that they would soon meet a person to work on a task with them and that this person had a history of schizophrenia (we were clear that this person would not have access to their scores on the SC-IAT). Participants met the confederate briefly before completing the pre-interaction materials, and then worked with him on the task. Both the experimenter and the confederate were blind to condition. The participants completed post-interaction dependent measures, and were probed for suspicion and debriefed. No participants withdrew after full disclosure.

#### Pre-interaction dependent measures

As in Study 1, these measures were administered after the manipulation but before the upcoming interaction. They were identical to Study 1 but with a change to the manipulation check to include negative self-affect (as a measure of compunction): we used an 8-item measure of negative self-affect (Monteith et al., 2002; Voils et al., 2002). Example items are “disappointed with myself” and “regretful” (α = 0.93). This measure was scored −3 (“strongly disagree”) to +3 (“strongly agree”).

Participants completed measures of intergroup anxiety; self-anxiety (α = 0.87), other-anxiety (α = 0.86), and generic intergroup anxiety (α = 0.90). They also completed two measures of expectancies; expectancy of self-efficacy (α = 0.80), and expectancy that the other is open to meeting with you (α = 0.88) as described in Study 1.

#### Post-interaction dependent measures

The post-interaction measures were identical to those used in Study 1. Participants rated how much they had enjoyed the interaction (α = 0.96) and were interested in future interactions (α = 0.92). They rated how friendly the confederate was (α = 0.65) and completed the 12 item stereotyping of the confederate measure (α = 0.90).

### Results and discussion

#### Manipulation check

Consistent with Monteith and colleagues, participants in the high compunction condition reported higher levels of negative self-affect compared to participants in the low compunction condition (*M*_compunction_ = −0.11, *SD* = 1.29; *M*_low compunction_ = −2.14, *SD* = 0.80; *p* < 0.001, *d* = 1.00, η*p*^2^ = 0.493; see Table 4). This indicated that the compunction manipulation was successful.

**Hypothesis 2**. We predicted that participants in the high compunction condition would experience more negative intergroup contact compared to participants in the low compunction condition. This was supported by our data (see Table 4). Participants in the high compunction condition reported that they had enjoyed the interaction less (*M*_high compunction_ = 5.31, *SD* = 0.89; *M*_low compunction_ = 6.11, *SD* = 0.77; *p* < 0.001, *d* = 0.957, η*p*^2^ = 0.191), were less interested in future interactions (*M*_high compunction_ = 5.81, *SD* = 0.92; *M*_low compunction_ = 6.40, *SD* = 0.66; *p* < 0.01, *d* = 0.801, η*p*^2^ = 0.121), thought that the confederate was less friendly (*M*_high compunction_ = 6.36, *SD* = 0.61; *M*_low compunction_ = 6.72, *SD* = 0.38; *p* < 0.01, *d* = 0.770, η*p*^2^ = 0.113), and stereotyped him more (*M*_high compunction_ = 2.29, *SD* = 0.70; *M*_low compunction_ = 1.74, *SD* = 0.66; *p* < 0.005, *d* = 0.877, η*p*^2^ = 0.146). The effect sizes were all medium to large.

**Hypothesis 4**. We predicted that participants in the high compunction condition would report lower expectancies of self-efficacy and higher levels of self-anxiety (compared to participants in the low compunction condition). Results supported these predictions (see Table 5). Participants in the high compunction condition reported more generic intergroup anxiety (*M*_high compunction_ = −0.55, *SD* = 1.16; *M*_low compunction_ = −1.26, *SD* = 1.12; *p* < 0.05, *d* = 0.659, η*p*^2^ = 0.090) and more self-anxiety (*M*_high compunction_ = −0.10, *SD* = 0.97; *M*_low compunction_ = −0.63, *SD* = 0.97; *p* < 0.05, *d* = 0.553, η*p*^2^ = 0.071). Participants also reported lower expectancy of self-efficacy (*M*_high compunction_ = 4.81, *SD* = 0.81; *M*_low compunction_ = 5.39, *SD* = 0.95; *p* < 0.05, *d* = 0.718, η*p*^2^ = 0.101). The effect sizes were all medium. There were no effects on other-anxiety or expectancy of the other.

**Table 4:** **Contrasting the contact and stereotyping dependent measures by condition (Study 2)**.

**Measure**	**Low compunction mean *(SD)***	**High compunction mean *(SD)***	
Manipulation check	−2.14 (0.80)	−0.11 (1.29)	*t*_(59)_ = 7.57, *p* < 0.001, *d* = 1.0, *ηp^2^* = 0.493
Enjoyed the interaction	6.11 (0.77)	5.31 (0.89)	*t*_(59)_ = 3.74, *p* < 0.001, *d* = 0.957, *ηp^2^* = 0.191
Interest in future interaction	6.40 (0.66)	5.81 (0.92)	*t*_(59)_ = 2.85, *p* < 0.01, *d* = 0.801, *ηp^2^* = 0.121
Friendliness	6.72 (0.38)	6.36 (0.61)	*t*_(59)_ = 2.74, *p* < 0.01, *d* = 0.770, *ηp^2^* = 0.113
Stereotyping	1.74 (0.66)	2.29 (0.70)	*t*_(59)_ = 3.17, *p* < 0.005, *d* = 0.877, *ηp^2^* = 0.146

**Table 5:** **Contrasting the expectancies and intergroup anxiety dependent measures by condition (Study 2)**.

**Measure**	**Low compunction M *(SD)***	**High compunction M *(SD)***	
Generic intergroup anxiety	−1.26 (1.12)	−0.55 (1.16)	*t*_(59)_ = 2.41, *p* < 0.05, *d* = 0.659, *ηp^2^* = 0.090
Self-anxiety	−0.63 (0.97)	−0.10 (0.97)	*t*_(59)_ = 2.13, *p* < 0.05, *d* = 0.553, *ηp^2^* = 0.071
Other-anxiety	−2.19 (0.78)	−1.79 (0.83)	*t*_(59)_ = 1.93, *p* = 0.059, *d* = 0.048, *ηp^2^* = 0.059
Expectancy of self-efficacy	5.39 (0.95)	4.81 (0.81)	*t*_(59)_ = 2.58, *p* < 0.05, *d* = 0.718, *ηp^2^* = 0.101
Expectancy of the other	5.95 (0.83)	5.77 (0.72)	*t*_(59)_ = 0.93, *p* = 0.36, *d* = 0.151, *ηp^2^* = 0.015

The study aimed to evaluate a compunction intervention on an actual intergroup interaction. Our results suggested that the high compunction intervention had negative effects on contact, stereotyping, self-efficacy, and self-intergroup anxiety. This latter is the form of intergroup anxiety which is specifically associated with anxiety about appearing prejudiced (either to the self or to the other person). The high compunction intervention seemed to cause participants to focus inwardly on themselves during the interaction (hence the effects on expectancies of self-efficacy and self-anxiety) rather than outwards toward their interaction partner (hence the null effects on expectancies of the other and other-anxiety).

## General discussion

Across two studies, we tested the separate effects of promotion (Study 1) and then compunction (Study 2) on participants' interactions with a confederate whom they believed to have a history of schizophrenia. Prejudice, stereotyping, and discrimination are driven by a number of processes operating at different levels, so it makes sense to explore how an intervention designed to tackle one process (e.g., unconscious bias) can impact on another (e.g., intergroup contact). Any intervention that can improve (or undermine) the success of intergroup contact is particularly relevant, because the success or failure of intergroup contact has profound effects on the experiences of both minorities and majorities (Graf et al., 2014). Contact is therefore a key area on which to focus. As has been noted elsewhere, experimental designs using real interactions are also surprisingly rare (Hebl and Dovidio, 2005). Our data therefore has a real world significance that is often absent from intergroup intervention research.

The results indicated a broadly positive effect of the promotion intervention. Working from the assumption that many participants enter intergroup interactions with avoidance motivations (i.e., to avoid thinking or doing something inappropriate), Trawalter and Richeson (2006) designed a simple intervention that instructed participants to relax and enjoy an interaction. Our data indicate that this intervention had medium to large effect sizes on participants' post-interaction feelings and stereotypes, with *ds* between 0.55 and 0.98 (see Cohen, 1988).

In contrast, the high compunction intervention had broadly negative effects (compared to low compunction). Monteith (1993) suggested that participants can be motivated to change by alerting them to the gap between how they should behave and how they actually behave. Our results suggest that there is not a simple relation between this motivation and the success of intergroup contact: participants in the high compunction condition reported lower levels of self-efficacy and increased levels of intergroup anxiety, as well as a less positive contact experience in general. These results need to be treated with some caution in the absence of a non-compunction control condition: it is possible that the difference between the high and low compunction conditions were driven by positive effects in the low compunction condition (rather than negative effects in the high compunction condition). Nevertheless, our data suggests that high levels of compunction are at best ineffective, and at worst potentially damaging to intergroup contact. Of course, the compunction intervention was not designed to promote positive intergroup contact, and so there is no reason to expect that it should have positive effects in this specific context. At the same time, however, there is evidence that negative contact experiences can have a disproportionately negative affect on prejudice (Barlow et al., 2012). Our results therefore suggest that compunction researchers should begin to explore how the undoubtedly positive effects of compunction can be extended into positive contact experiences.

One key limitation in our design relates to generalization. Although participants in the promotion condition had more positive contact experiences, and stereotyped their interaction partner less, we have no data on how these effects might generalize to the target group as a whole (i.e., people who have a history of schizophrenia) or on future intergroup contact (Paolini et al., 2014). We also have no data on how enduring these effects are over time (see also Paolini et al., 2016). Pettigrew (1998) suggested that it was important to consider the sequence of interventions over time. We suggest that the same approach be taken to promotion and compunction: low to moderate levels of compunction might be an effective early intervention that motivates participants to engage, while promotion during contact reduces participants' avoidance motivations and thereby facilitates good contact.

## Ethics statement

This study was carried out in accordance with the recommendations of APA guidelines. Participants gave written informed consent in accordance with the Declaration of Helsinki. They were also given the opportunity to withdraw after debriefing and full disclosure. The protocol was approved by the Cardiff School of Social Sciences Research Ethics Committee.

## Author contributions

Conceptualization: KG, GM; Data Curation: KG, DX; Formal Analysis: KG, DX; Funding Acquisition: KG, GM; Investigation: DX; Methodology: KG, GM; Project Administration: DX; Software: DX; Supervision: KG, GM; Writing—Original Draft Preparation: KG, DX, GM; Writing—Review and Editing: KG, GM.

## Funding

This research was funded by the UK Economic and Social Research Council grant number RES000231106.

### Conflict of interest statement

The authors declare that the research was conducted in the absence of any commercial or financial relationships that could be construed as a potential conflict of interest.
