# Supplementary material for: Effects of Promotion and Compunction Interventions on Real Intergroup Interactions: Promotion Helps but High Compunction Hurts
Source: Front Psychol. 2017 Apr 7;8:528. doi: 10.3389/fpsyg.2017.00528 (PMC5383699; doi:10.3389/fpsyg.2017.00528)
Supplement: Supplementary file 1 [file DataSheet1.docx]

# Appendix 1

SC-IAT stimulus materials

| Mental illness words | Good words* | Bad words* |
| --- | --- | --- |
| Barmy | Beautiful | Angry |
| Crazy | Celebrating | Brutal |
| Insane | Cheerful | Destroy |
| Mad | Excellent | Dirty |
| Mental | Fabulous | Disaster |
| Psycho | Friendly | Disgusting |
| Schizo | Glad | Dislike |
| Screwy | Happy | Evil |
| Whacko | Likable | Gross |
|  | Loving | Horrible |
|  | Marvellous | Humiliate |
|  | Pleasure | Nasty |
|  | Smiling | Painful |
|  | Splendid | Revolting |
|  | Superb | Sickening |
|  | Paradise | Terrible |
|  | Triumph | Ugly |
|  | Wonderful | Unpleasant |

* evaluative words taken from Karpinski and Steinman (2006)
